# Geopolymer/CeO_2_ as Solid Electrolyte for IT-SOFC

**DOI:** 10.3390/polym12010248

**Published:** 2020-01-20

**Authors:** Jelena Gulicovski, Snežana Nenadović, Ljiljana Kljajević, Miljana Mirković, Marija Nišavić, Milan Kragović, Marija Stojmenović

**Affiliations:** 1 Laboratory for Materials Sciences, Institute of Nuclear Sciences Vinča, University of Belgrade, 11000 Belgrade, Serbiam.kragovic@vinca.rs (M.K.);; 2 Laboratory of Atomics Physics, Institute of Nuclear Sciences Vinča, University of Belgrade, 11000 Belgrade, Serbia

**Keywords:** polymer composites, geopolymers/ceria, electrical conductivity, fuel cells

## Abstract

As a material for application in the life sciences, a new composite material, geopolymer/CeO_2_ (GP_CeO_2_), was synthesized as a potential low-cost solid electrolyte for application in solid oxide fuel cells operating in intermediate temperature (IT-SOFC). The new materials were obtained from alkali-activated metakaolin (calcined clay) in the presence of CeO_2_ powders (x = 10%). Besides the commercial CeO_2_ powder, as a source of ceria, two differently synthesized CeO_2_ powders also were used: CeO_2_ synthesized by modified glycine nitrate procedure (MGNP) and self-propagating reaction at room temperature (SPRT). The structural, morphological, and electrical properties of pure and GP_CeO_2_-type samples were investigated by X-ray powder diffraction (XRPD), Fourier transform infrared (FTIR), BET, differential thermal and thermogravimetric analysis (DTA/TGA), scanning electron microscopy (FE-SEM), energy dispersive spectrometer (EDS), and method complex impedance (EIS). XRPD and matrix-assisted laser desorption and ionization time-of-flight (MALDI-TOF) analysis confirmed the formation of solid phase CeO_2_. The BET, DTA/TGA, FE-SEM, and EDS results indicated that particles of CeO_2_ were stabile interconnected and form a continuous conductive path, which was confirmed by the EIS method. The highest conductivity of 1.86 × 10^−2^ Ω^−1^ cm^−1^ was obtained for the sample GP_CeO_2__MGNP at 700 °C. The corresponding value of activation energy for conductivity was 0.26 eV in the temperature range 500–700 °C.

## 1. Introduction

Due to high efficiency, long-term stability, low emissions, and relatively low cost, solid oxide fuel cells (SOFCs) present promising electrochemical conversion devices. This class of fuel cells can use various fuels, such as hydrogen, biogas, methane, and natural gas, with very limited emission levels [1,2,3,4]. The largest deficiency of these cells is the high operating temperature of approximately 1000 °C. High operating temperatures result in different issues, such as long start-up/shutdown times, thermal stress, and finally the material degradation [3,4]. However, with decreases of the operating temperature below 1000 °C, the SOFC cell performance decreases due to an increase in the internal resistance. The factors causing the increase in the internal resistance can be the high resistance of electrolyte, low conductivity, and high polarization resistance of cathode. To obtain a satisfactory performance of SOFC at reduced operating temperatures, it is necessary to develop a solid electrolyte with rather high efficiency and conductivity which is operational in the intermediate temperature range 500–700 °C (IT-SOFC) [5,6].

Keeping these requirements in mind, materials based on CeO_2_ are the most appropriate candidates as solid electrolytes. Due to its unique properties, such as the excellent absorption of UV radiation, high stability at high temperatures, a large oxygen storage capacity, high hardness, and high reactivity, CeO_2_ is used also in various fields of science and industry [7,8,9,10,11,12,13]. Among a number of different methods for producing nanoscaled ceria, use of the modified glycine–nitrate procedure (MGNP) and self–propagating reaction at room temperature (SPRT) present two of the most promising methods for the synthesis of CeO_2_ nanopowders [12,13]. The fluorite-type crystal lattice of CeO_2_ allows replacing a relatively large amount of cerium cations by M cations (e.g., group IIA or rare earth metals), which can significantly influence its conductivity [14,15]. The substitution of Ce^4+^ cations with trivalent cations introduces oxygen vacancies because of charge compensation. The existence of vacancies enables the movement of the oxygen anions, i.e., the transport of oxygen from the cathode to the anode in SOFC. The possibility of releasing a large number of oxygen atoms from the crystal lattice (reduction) and re-filling with new O atoms (oxidation) is the most important property required of the fuel cell material. These processes should be rapid and highly reversible, so that reduction and oxidation processes can be carried out several times. However, the problem of CeO_2_ is a very high cost price, which requires a progress in the development of lower cost material as a new generation of solid electrolyte for IT-SOFC.

In this context, with the advances in ceramic processing technology, ceramic–matrix composites have been increasingly used in recent years [16,17]. Good examples for that are multidoped calcium phosphate and alumina/YAG [16,17], or aluminosilicates [18]. The aluminosilicate ceramica in form of a geopolymer (GP) represent one of the few alternatives as a potential low-cost material for solid oxide fuel cell applications [18]. Geopolymer is a relatively new alumosilicate inorganic cross-linked long-chain polymer material, which has excellent properties such as high strength, anticorrosion, standing fire, and long life. The typical geopolymer composition is expressed as nM_2_O·Al_2_O_3_·xSiO_2_·yH_2_O (M is an alkali metals element such as Li, Na, or K) [19]. The molecular structure of geopolymers is described as a matrix of alumina and silica tetrahedra joined at the corners with oxygen [20]. In the geopolymer materials, water molecules and hydroxyl groups are the most important factors that influence the electrical conductivity and dielectric property at room temperature, but this effect can be eliminated if the geopolymer materials are treated at high temperature, as shown in the investigation of Cui et al. [19]. In addition, the modification of aluminosilicate ceramics by adding some elements, such as Mg in small amounts, leads to a highly developed specific area (microcrystalline and amorphous regions of stoichiometric and nonstoichiometric oxides), with certain porosity in form of highly developed metallized surfaces [21]. Adding elements such as Mg, Ni, Ti, or graphite, in small amounts, allows the deposition of the micro and nanostructured films and clusters, which play an essential role in ion-electron processes and microgalvanic couples, through which the transport of charges and conductivity is carried out [21,22,23]. This modification process enables obtaining structurally modified multifunctional materials with electrochemical and electrophysical activities [22,23]. That implies the generation of microgalvanic couples and certain solid phases that make aluminiumsilicate ceramics active. The appearance of microgalvanic couples and ion-electron processes through formed solid phases present additional micromorphology secondary changes [24]. In contrast to perovskite and fluorite, in which oxide ion conduction proceeds via oxygen vacancies [6,14,15,16,17,18,19,20], the research of geopolymer materials suggests that conductivity involves interstitial or free ions [6]. Recent papers show a wide range of cation substitutions due to the flexibility of the structure in accommodating a range of ion sizes [25,26,27]. In addition, studies on doped samples [28] show achieving high conductivity, non-stoichiometry in terms of cation vacancies, and/or oxygen excess requirements [29]. The optimization of geopolymer systems, particularly in terms of adding different elements (such as rare earths elements) or electrolyte systems (multidoped oxides of rare earths elements) at low concentrations improve conductivity, which leads to the application of geopolymer-type electrolytes in IT-SOFC.

The main aim of this paper is the addition of different CeO_2_ powders (commercial powder (CeO_2__com), powders obtained by MGNP (CeO_2__MGNP), and SPRT methods (CeO_2__SPRT)) in inorganic polymer–geopolymer (GP), in order to develop new composite materials (composite geopolymer with CeO_2__com (GP_CeO_2__com), with CeO_2__MGNP (GP_CeO_2__MGNP), and with CeO_2__SPRT (GP_CeO_2__SPRT)), as solid electrolytes for application in IT-SOFC. The application of newly formed electrolytes in intermediate temperature solid fuel cells announces the development of clean and inexpensive technology, with the characteristics of direct conversion of energy to electricity, featuring high efficiency and reliability.

## 2. Materials and Methods 

### 2.1. Samples Preparations

In order to develop new composite materials, pure GP, GP_CeO_2__com, GP_CeO_2__MGNP, and GP_CeO_2__SPRT as solid electrolytes, were synthesized. In the first step, metakaolin (MK) in form of pure GP was prepared. After that, three new mixtures of GP with 10 mass% of three differently synthesized CeO_2_ powders were made. Metakaolin (MK) was prepared by the calcination of kaolinite at 750 °C at a constant heating rate of 10 °C/min with a hold time of 3 h [30]. The new materials were obtained by alkali-activated metakaolin (calcined kaolin) in the presence of CeO_2_ powders. Beside the commercial CeO_2_ powder (Sigma Aldrich, 99.999%, Merck KGaA, Darmstadt, Germany), as a source of ceria, two differently synthesized CeO_2_ powders were also used: CeO_2_ synthesized by the MGNP procedure (particle size about 40 nm) and SPRT method (particle size about 5 nm). [31,32,33]. All mixtures were alkali activated by sodium silicate (Na_2_SiO_3_) and 12 M NaOH (Sigma Aldrich analytical grade, Merck KGaA, Darmstadt, Germany) (solid/liquid = 0.85). The samples were cast into molds, and all geopolymers (GP, GP_CeO_2__com, GP_CeO_2__MGNP and GP_CeO_2__SPRT) were formed after incubating at room temperature for one day, and at 50 °C for 2 days in a sample drying oven. After removal from the molds, the samples were processed to the dimensions necessary for characterization by the different methods presented in the following sections. 

### 2.2. Methods

All of the produced geopolymers–CeO_2_ samples were characterized by X-ray powder diffraction (XRPD), Fourier-transform infrared (FTIR), and thermal analysis (DTA/TGA), as well as with scanning electron microscopy (FE-SEM), energy dispersive spectrometer (EDS), matrix-assisted laser desorption (MALDI), and complex impedance methods (EIS). 

For X-ray powder diffraction analysis, an Ultima IV Rigaku diffractometer, equipped with CuKα1,2 radiation, using a generator voltage (40.0 kV) and a generator current (40.0 mA), at room temperature was used. The range of 5–80° 2θ was used for all powders in a continuous scan mode with a scanning step size of 0.02° and at a scan rate of 2°/min. Phase identification of samples were performed using PDXL2 software (version 2.8.3.0.) and ICDD database [34,35].

Fourier-transform infrared (FTIR) spectra of the investigated samples were collected using a Thermo Fisher Scientific FTIR spectrometer IS-50 in the transmission mode. The samples were prepared by using pressed KBr pellets (0.2 mg of the sample: 80 mg KBr) technique. The spectra were recorded in the range from 4000 to 400 cm^−1^ with 64 scans and resolution of 2 cm^−1^. After recording of the spectra, two corrections were performed: automatic correction of the base line and atmospheric suppression.

The specific surface area and the pore size distribution (PSD) of all the investigated samples were analyzed using the Surfer (Thermo FisherScientific, Houston, Texas, USA). PSD was estimated by applying the Barrett–Joyner–Halenda (BJH) method to the desorption branch of the isotherms and mesopore surface, while micropore volumes were estimated using the α_s_–plot method [36]. The values of the micropore surface were calculated by subtracting the mesopore surface from the total specific surface area [36,37,38,39].

Matrix-assisted laser desorption and ionization time-of-flight (MALDI-TOF) mass spectra were acquired on a Voyager DE ProWorkstation (Perseptive Biosystems, Framingham, MA, USA). The system utilizes a 20 Hz pulsed nitrogen laser emitting at 337 nm. The spectra were acquired without a low mass gate and under delayed extraction conditions in the reflector mode to increase the mass accuracy and resolution, respectively. The spectra were acquired in the positive ion mode, without a matrix (LDI mode). A small volume of each sample (0.5 µL) was applied on the MALDI target plate followed by the application of the small volume of the matrix solution (if applied). The mixture was left afterwards on the room temperature to dry. 

Thermal analyses (thermogravimetric (TGA) and differential thermal analysis (DTA)) of investigated samples were performed on a Netzsch STA 409 EP. Samples were heated from 25 to 1000 °C in an air atmosphere at the heating rate of 10 °C/min. Prior to analysis, samples were kept in a desiccator at a relative humidity of 23%.

The morphology and microstructure of the all samples were studied by field emission scanning electron microscopy (FESEM) TESCAN Mira3 XMU at 20 kV. The samples were precoated with a layer of gold before observation that was several nanometers thick. The images were recorded at a magnification ×100 kx with an accelerating voltage of 20 kV. The chemical compositions of the GP_CeO_2_ samples were analyzed by an energy dispersive spectrometer (EDS) Isis 3.2, with a SiLi X-ray detector (Oxford Instruments).

Shortly after synthesis, the electrical properties of the obtained samples were measured by the complex impedance method, in a frequency range of 10 µHz to 1 MHz, using Interface 1000 Potentiostat/Galvanostat/ZRA and EIS300 Electrochemical Impedance Spectroscopy Software. The measurements were conducted in air, in the temperature range of 500–700 °C, with 50 °C increments. The amplitude of the applied sinusoidal voltage signal was 20 mV. A thin layer of high conductivity silver paste was applied onto both sides of the sample pellets (8 mm × 2.5 mm) in order to provide good electrical contact between the electrolyte and electrodes. The samples were placed between the silver plates in a ceramic holder that was heated by vertical oven. A Pt–Rh thermocouple located just below the bottom silver plate was used for temperature monitoring. The impedance plots obtained experimentally were fitted by means of the software ZViews for Windows (Version3.2b). The resistance values were determined from the impedance diagrams recorded at various temperatures. The specific conductance was calculated from the resistance data using the dimensions of the sample pellets. 

## 3. Results and Discussion

### 3.1. XRPD Analysis

**Figure 1:**
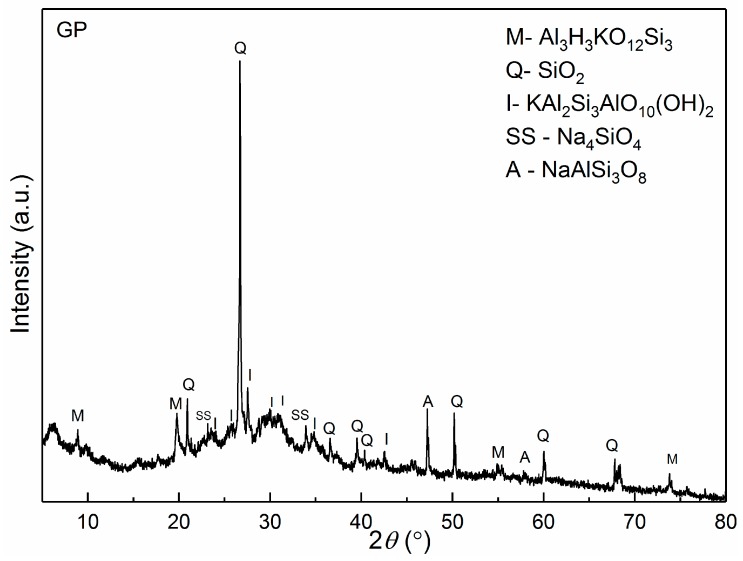
X-ray diffraction pattern of pure geopolymer (GP) sample.

**Figure 2:**
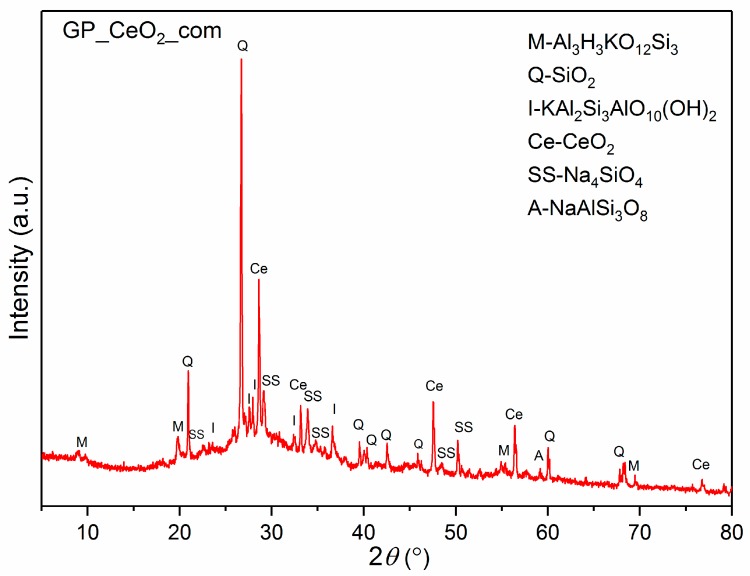
X-ray diffraction pattern of a GP_CeO_2__com sample.

**Figure 3:**
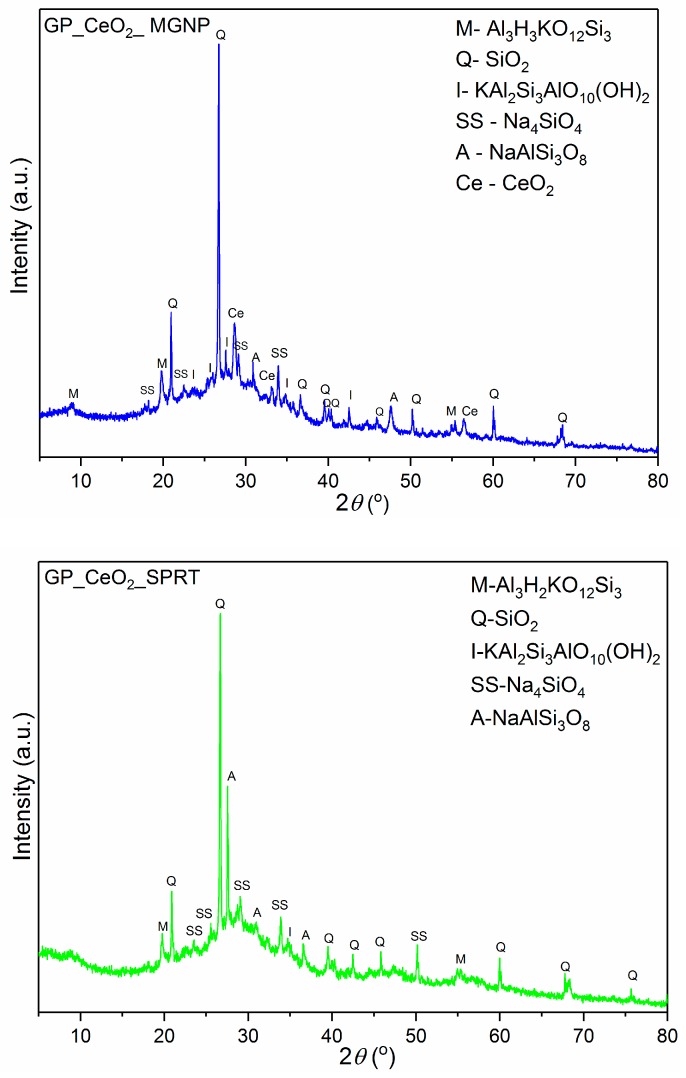
X-ray diffraction patterns of: (**a**) GP_CeO_2__MGNP and (**b**) GP_CeO_2__SPRT samples.

The mineralogical compositions of all the obtained samples were determined by XRPD analysis (Figure 1, Figure 2 and Figure 3). Figure 1 shows the diffraction pattern of the synthesized GP. This material is also used as the initial material for CeO_2_-based geopolymers. It is evident that the phase composition corresponds to geopolymers synthesized from metakaoline as the primary raw material, as described in previous papers [40,41]. In addition, from a diffractogram of pure GP, it is observed that the mineral phases SS–Na_4_SiO_4_ (ICDD 00-032-1154) was formed during the synthesis of sodium silicate by using a high concentration of NaOH (14 M) and silicon glass. From Figure 1, it is clearly visible that quartz (Q–SiO2; ICDD 01-079-1970) was dominate in crystal phase of pure GP, while the content of other minerals—illite (I: KAl_2_Si_3_AlO_10_(OH)_2_; ICDD 00-002-0056), muscovite (M: Al_3_H_2_KO_12_Si_3_; ICDD 00-002-0055), and albite (A: NaAlSi_3_O_8_; ICDD 01-071-1154) were significantly lower.

The XRPD pattern of the GP_CeO_2__com sample is shown in Figure 2. From the presented diffractogram, the peaks of CeO_2_ phase are visible (ICDD 00-004-0593). Even more, addition of the ceria to the system has led to increasing sodium silicate phases and decreasing aluminum and potassium phases (muscovite and illite). The high background, especially in range of 20–50 2θ(°), most likely originated from amorphous silica.

In Figure 3, powder diffraction patterns of geopolymer samples synthesized with CeO_2_ obtained by MGNP and SPRT methods are shown. The pattern of the GP_CeO_2__MGNP sample shows the presence of ceria peaks, and it is very similar to the diffractogram of GP_CeO_2__com. On the other hand, from the diffraction pattern of the GP_CeO_2__SPRT sample, the absence of peaks that are related to ceria is evident. The abandonment of these peaks indicates an inability to dissolve the ceria powder in the geopolymer matrix in a highly alkaline environment. The assumption is that the ceria nanoparticles that were obtained by the SPRT method most likely were trapped in the silicate matrix of the geopolymer. The mineral composition of the obtained sample was very similar in comparison with previous patterns of the GP_CeO_2__MGNP sample. The well-defined peak of albite around 27.51 °2θ leads to the assumption that the preferred orientation of albite crystals is achieved in sample preparation.

### 3.2. FTIR Analysis

**Figure 4:**
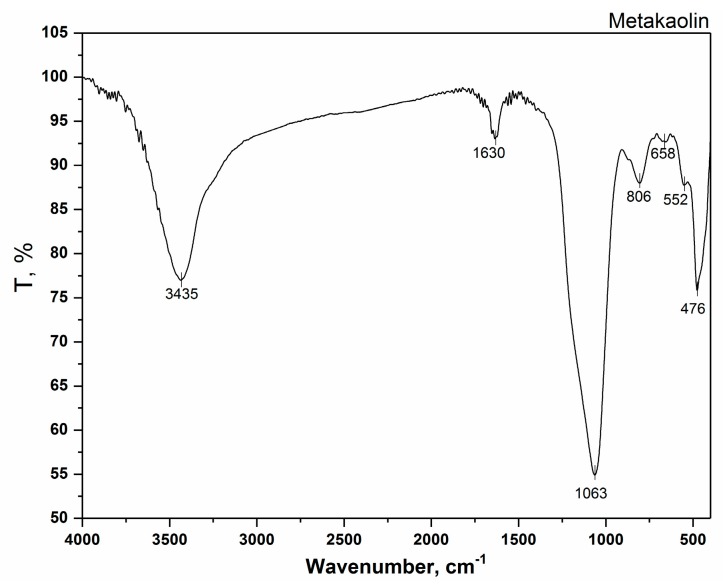
Fourier transform infrared (FTIR) spectrum of the metakaolin.

The result of the FTIR analysis of the metakaolin is presented in Figure 4. In the FTIR spectrum of metakaolin, seven clearly expressed bands are observed. The band at 3435 cm^−1^ may be attributed to stretching vibrations, while the band at 1630 cm^−1^ originated from the bending vibrations of the water –OH groups. Spectral bands in the range from 1100 to 400 cm^−1^ originate from the structural vibrations of the aluminosilicate metakaolin. Thus, the band at 1063 cm^−1^ may be attributed to the asymmetric stretching vibrations of the Si (Al)–O groups. Absence of the band at 539 and 913 cm^−1^ characteristics for kaolin and the appearance of a new band at 806 cm^−1^ may be related to the change from the octahedral coordination of Al^3+^ in kaolinite to the tetrahedral coordination in metakaolinite [42]. The band at 658 cm^−1^ is due to the symmetric vibrations of the Si (Al)–O groups. The band at 552 cm^−1^ originates from double ring vibrations, while the band at 476 cm^−1^ is derived from the bending vibrations of the SI (Al)–O group.

**Figure 5:**
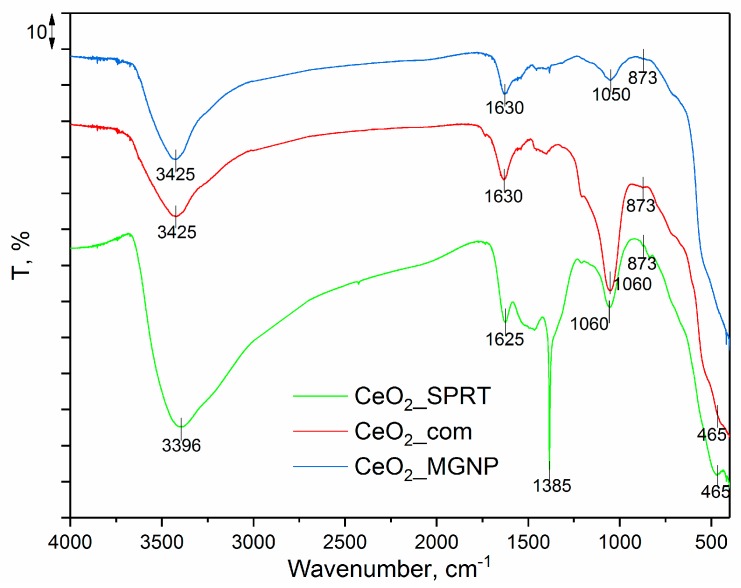
FTIR spectra of the CeO_2__com, CeO_2__MGNP, and CeO_2__SPRT samples. MGNP: modified glycine nitrate procedure, SPRT: self-propagating reaction at room temperature.

In the Figure 5, the spectra of the commercial CeO_2_ powder as well as the spectra of CeO_2_ synthesized by a modified glycine nitrate procedure and self-propagating reaction at room temperature are presented. In spectrum of the commercial CeO_2_ powder, two bands at 3425 and 1630 cm^−1^ are ascribed to the O–H stretching vibrations in OH– groups and represent the water presented in the investigated sample [43]. The bands at 1060, 873, and 465 cm^−1^ are characteristic for CeO_2_ and may be attributed to Ce–O vibrations [44].

In the spectra of the CeO_2__MGNP and CeO_2__SPRT, all the spectral bands that are characteristic for commercial CeO_2_ are visible with the only difference in the spectrum of the CeO_2__SPRT in the form of the sharp intensive peak at 1385 cm^−1^, which corresponds to the N–O antisymmetric stretching of the residual nitrate groups, ν(NO_3_^−^) [45,46].

**Figure 6:**
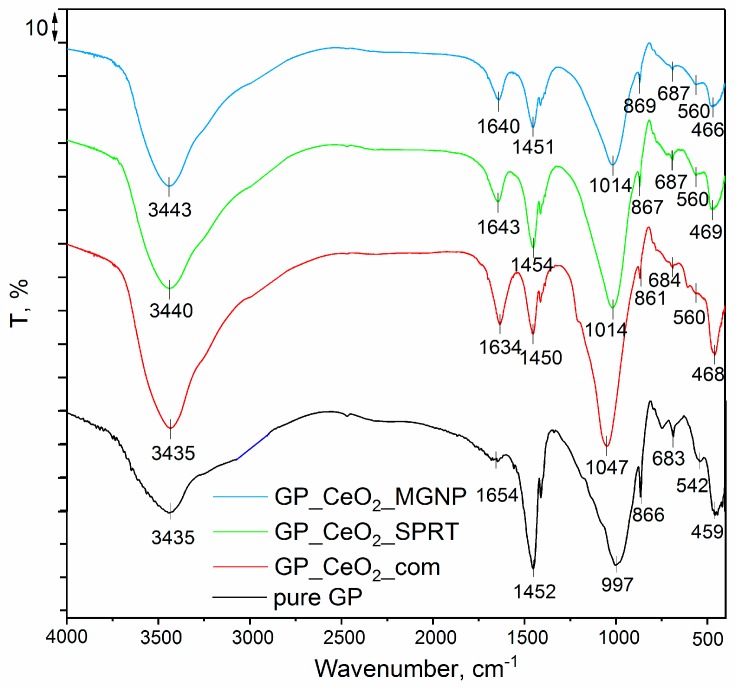
FTIR spectra of the pure GP, GP_CeO2_com, GP_CeO2_MGNP, and GP_CeO2_SPRT samples.

The structural properties of the materials obtained by activation of the metakaolin (calcined kaolin) with sodium silicate (Na_2_SiO_3_) and 12 M NaOH without the presence of the CeO_2_ (pure GP), as well as in the presence of CeO_2_ (CeO_2__com, CeO_2__MGNP, and CeO_2__SPRT) were also investigated, and the results are given in Figure 6. As it may be seen, after comparison of the results presented in Figure 6 with those presented in Figure 4, all the spectral bands of the metakaolin are visible in the spectra of pure GP, GP_CeO_2__com, GP_CeO_2__MGNP, and GP_CeO_2__SPRT. In addition, the spectral bands of CeO_2_ are visible without significant changes in their positions in the GP_CeO_2__com, GP_CeO_2__MGNP, and GP_CeO_2__SPRT samples. On the other side, due to the overlapping bands characteristic for metakaolin and CeO2, increasing of the band intensities at about 1000 cm^−^^1^ in the spectra of the GP_CeO_2__com, GP_CeO_2__MGNP, and GP_CeO_2__SPRT is visible. Due to the thermal decomposition reactions of Ce(NO_3_)_3_, which occurred during the synthesis of the GP_CeO_2__SPRT composite, this peak is not observed on the spectrum of GP_CeO_2__SPRT (Figure 6). The existence of all the spectral lines characteristic for the starting samples (metakaolin and CeO_2_) without significant changes in their positions indicate that the applied activation procedure did not cause a significant change in the structure of the starting samples. The band at approximately 1400 cm^−1^ is the only difference in the spectra of the activated and initial samples. Heah et al. used a mixture of NaOH and sodium silicate solutions for the production of alkali-activated kaolin [47]. The authors also found appearances of the spectral band at about 1400 cm^−1^ after the activation process and assigned it to the Si–O–Si stretching vibrations. The authors explained that the higher energy necessary for the Si–O–Si vibrations in comparison with the Si–O–Al vibrations explained the increasing of the compressive strength of the samples after the activation and presence of NaOH.

### 3.3. MALDI TOF Analysis

**Figure 7:**
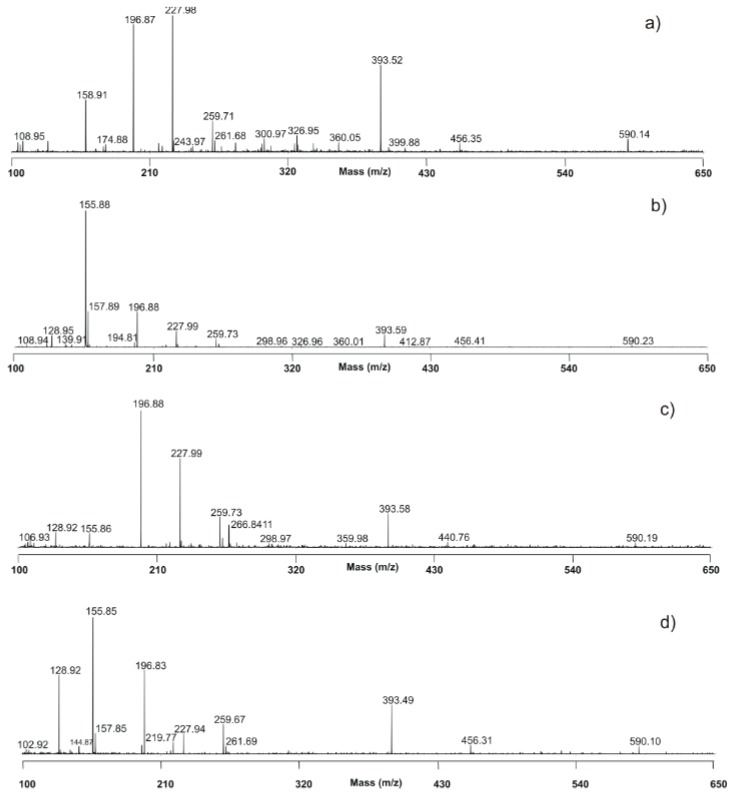
Matrix-assisted laser desorption and ionization time-of-flight (MALDI-TOF) analysis of (**a**) GP, (**b**) GP_CeO_2__com, (**c**) GP_CeO_2__SPRT and (**d**) GP_CeO_2__MGNP.

Figure 7 represents results obtained using LDI (without matrix). The laser-excited geopolymer sample provided the following characteristics peaks at m/z = 197.0, 228.0, 259.7, 393.6, 456.3, and 590.2. According to the literature data, these peaks corresponds to AlSi_2_O_5_(OH)^2+^, Al_2_Si_2_O(OH)^6+^, ((NaO)_4_SiO_2_O_3_)^2−^, (AlSi_2_O_5_(OH)_2_)^2+^, (Al_2_Si_2_O(OH)_6_)^2+^, and (AlSi_2_O_5_(OH)_2_)^3+^, respectively (Figure 7a–d) [48]. The identity of signals detectable at m/z = 156 and 157 corresponds to the CeO^+^ and CeOH^+^ ions (Figure 7b–d) [48]. Regarding the geopolymer, the peaks obtained at m/z = 393.6 and 591 corresponds to the dimer and trimer from the AlSi_2_O_5_(OH)^2+^ monomer, while the peak obtained at m/z = 456.3 belongs to the dimer from the Al_2_Si_2_O(OH)^6+^ monomer. This definitely favors the polymerization process. When it comes to CeO_2_, the characteristic peaks such as m/z = 445.7 for Al_2_Ce_2_O_7_ or m/z = 447.7 for Ce_2_Si_2_O_7_ have not been found, so we can not say that the cerium is chemically bound to the GP, but according to XRPD analysis, we assume that it is trapped in the so-called cages in the form of a second solid phase.

### 3.4. Adsorption isotherms—BET Analysis

**Figure 8:**
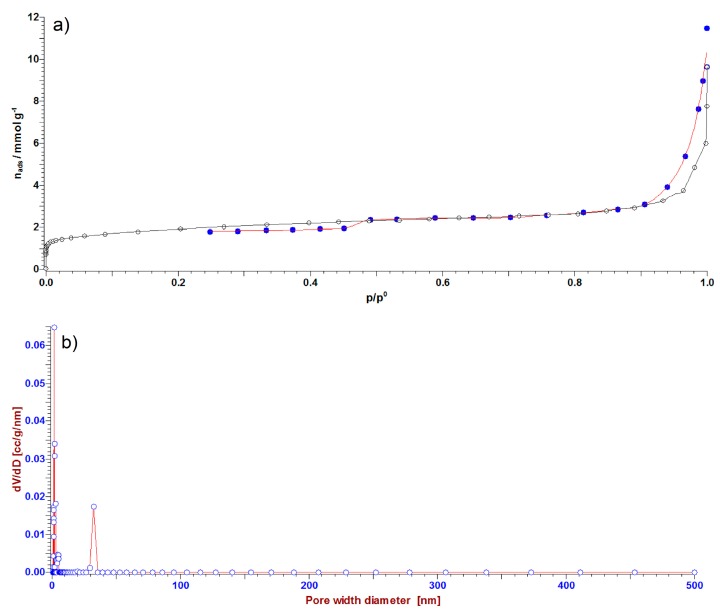
(**a**) Nitrogen adsorption isotherm of pure GP, given as the amount of N_2_ adsorbed as the function of a relative pressure, and (**b**) corresponding pore size distribution (PSD).

Nitrogen adsorption isotherms as the amount of N_2_ adsorbed in a function of relative pressure at −196 °C were determined for all geopolymers. According to the IUPAC (International Union of Pure and Applied Chemistry) classification, a nitrogen adsorption isotherm of pure GP was the type IV with a poor defined hysteresis loop (Figure 8a) [49]. The total surface area of the pure GP calculated by the BET equation (S_BET_) was 47.21 m^2^ g^−1^. The pore size distribution (PSD) presented in Figure 7b shows the presence of both micropores and mesopores. The calculated porosity parameters obtained using the α_s_-plot method show that the sample is dominantly mezoporous (S_meso_ = 30.55 m^2^ g^−1^) with a certain amount of microporosity (S_micro_ = 16.66 m^2^ g^−1^ and V_micro_ = 0.0078 cm^3^ g^−1^), which is in accordance with the PSD data. Generally, it is considered that the mesopores are formed between the particles, while micropores are formed on the particles [33]. Since the XRPD analysis showed that the particles of CeO_2_ have small diameters (order of magnitude nm) [31], during the agglomeration, they could be incorporated in micro and mesopores. This is supported by the results obtained by the BET method, in which samples of GP doped by different amounts of CeO_2_ have very small values of surface areas that are almost negligible. In this case, the formation of CeO_2_ as a second solid phase (confirmed by XRPD and MALDI-TOF MS analysis) presents an additional micromorphology change, whose geopolymer structure makes it electrochemically active (Session 3.7).

### 3.5. TGA-DTA Analysis

**Figure 9:**
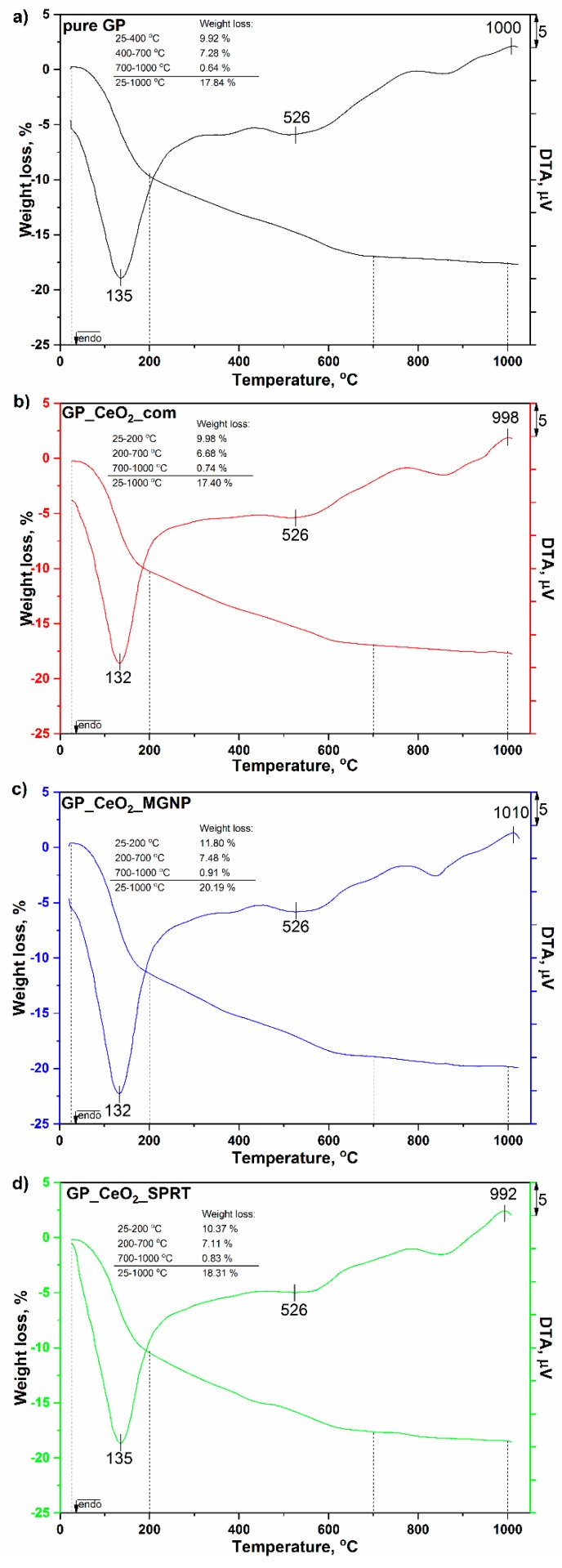
Results of the DTA/TGA analyses of: (**a**) GP, (**b**) GP_CeO_2__com, (**c**) GP_CeO_2__MGNP, and (**d**) GP_CeO_2__SPRT.

Results of the thermogravimetric (TG) and differential thermal analysis (DTA) of the pure GP and GP_CeO_2_ samples are given in Figure 9. As it may be seen from Figure 9, all the activated samples that contain cerium oxides show DTA/TGA diagrams that are similar with those that originated from the pure GP. The absence of the peaks characteristic for CeO_2_ may be explained with good thermal stability of the different CeO_2_ samples in geopolymer systems [50]. Owing to the stability of the fluorite type of crystal structure, the CeO_2_ shows a single phase and could possibly be used at high temperatures (over 1000 °C) without changing the structure. Thus, it can be concluded that ceria, as well as a geopolymer/CeO_2_ solid electrolyte, were characterized by high thermal stability, which is important for its potential application as a solid electrolyte for IT-SOFC. Accordingly, in the DTA diagrams of the investigated samples, there are only visible peaks characteristic for the metakaolin. In the temperature range 25–200 °C, an endothermic peak at approximately 132 °C was obtained in all the investigated samples. Those peaks originated from the release of the physically adsorbed water in pores and on the surfaces of the GP_CeO_2__MGNP. That was followed with adequate weight losses in the TGA diagrams in the same temperature interval (approximately 10% for GP_CeO_2__SPRT and approximately 12% for GP_CeO_2__MGNP). The endothermic peaks at 526 °C for all the samples originated from releasing the structural and stronger bound water from samples, and that process has been followed on the TGA diagram with weight losses of about 7% in the second temperature range (200–700 °C) for all the samples. The exothermic DTA peaks at about 1000 °C are characteristic for high-temperature phase transformation of metakaolin into mullite, which was not accompanied with a significant change in the mass of the tested samples on TGA curves (lower than 1% for all) [42].

### 3.6. SEM Analysis 

**Figure 10:**
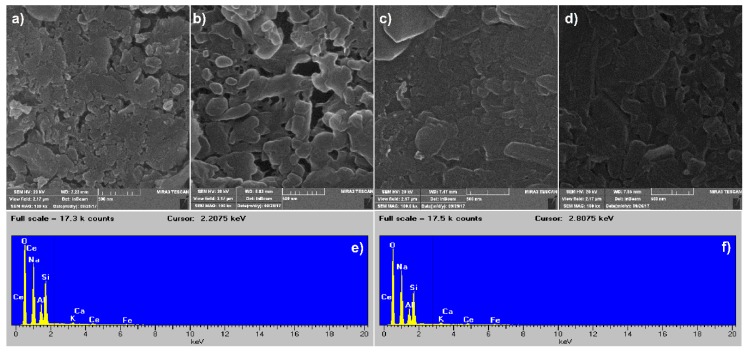
SEM images of: (**a**) GP, (**b**) GP_CeO_2__com, (**c**) GP_CeO_2__ SPRT, (**d**) GP_CeO_2__ MGNP, (**e**) and (**f**) corresponding energy dispersive spectrometer (EDS) spectra of grains and grain boundary GP_CeO_2__MGNP.

Throughout the process of geopolymer formation, there are several phases present in the system. These phases include unreacted particles, partially reacted particles, a newly formed (alumino)silicate gel, smaller (alumino)silicate species that have been liberated from the network, dissolved alkali metal hydroxides, and water [51]. Similar to the SEM results present in the literature [51], the obtained SEM images showed that the pure GP, as well as geopolymer/CeO_2_ samples (Figure 10), contained undisclosed calcined clay, some quantity of newly formed aluminosilicate species, and particles of irregular shape. As can be observed, pure GP (Figure 10a) and GP_CeO_2__com (Figure 10b) have sponge-like and coarse texture surfaces, while there is a homogeneous gel phase between the irregularly shaped particles. Namely, with increasing temperature, the gel phase formation occurs between particles, creating a homogeneous solid phase. A similar observation was reported by Ogundiran and Kumar [52] for metakaolin alkali sodium silicate geopolymers. Since active geopolymers have dominant amorphous microstructures and micromorphologies with perturbed crystal structures, adding elements leads to the formation of some active centers and potential barriers [21,24]. Therefore, the incorporation of CeO_2_ in micro and mesopores enables obtaining geopolymers–CeO_2_ with active local places in the form of a new solid phase. According to SEM images, in this way, a dense geopolymer structure was developed (Figure 10c,d). The results of the EDS analysis of the grains and grain boundary of the GP_CeO_2__MGNP (Figure 10e,f) showed the presence of the Ce at the surface of the investigated sample. This can be an indication that the micropores and mesopores of GP are filled with CeO_2_ nanoparticles, which probably are stabile interconnected, and that it is thus created a continuous conductive path.

Namely, as can be observed in Figure 10a–d, all the GP_CeO_2_ materials pose microstructures with different grain sizes (between 0.3 and 0.7 μm) and densities. Addition of the CeO_2_ with different particle sizes (CeO_2__com about 35 nm; CeO_2__MGNP about 40 nm [31]; CeO_2__SPRT about 5 nm [31]) leads to different filling of micropores and mesopores of pure geopolymer. The characterization process showed that CeO_2_ particles obtained by MGNP and SPRT had more uniform particles (good ordered structure), which led to better packing and a lower inner activity of particles (Figure 10c,d). That enabled forming a dense geopolymer/CeO_2_ structure, which is visible at Figure 10c,d. On the other hand, the CeO_2_ particles obtained by the commercial method possess non-uniform agglomerated particles with many vacancies. Such particles showed poor parking in micropores and mesopores and higher inner activity; therefore, lower density was expected (Figure 10b). The grain boundary (white parts) and grain of all the GP_CeO_2_ samples from Figure 10b–d were analyzed by the EDS method (Figure 10e,f). The EDS results of the grains and the grain boundary GP_CeO_2__MGNP reveal that both contain Si, Al, Na, K, Ca, Fe, Ce, and O. The atomic ratio of grains and grain boundary GP_CeO2_MGNP sample is Si/Al/Na/K/Ca/Fe/Ce/O = 23.33/12.81/17.58/0.82/0.62/0.15/6.61/38.08 and Si/Al/Na/K/Ca/Fe/Ce/O = 22.01/12.42/17.43/1.44/1.13/0.14/12.26/33.08. Based on these results, we can conclude that the CeO_2_ phase is more concentrated in the region of the grain boundary than in the grain itself. Thus, this CeO_2_ phase is white or at least brighter than the geopolymers in Figure 10b–d and makes the geopolymer/CeO_2_ materials electrochemically active.

### 3.7. Electrical Conductivity

The literature data showed that the complex impedance method gives great opportunities to study the electrical properties of solid materials [25,26,27]. Apart from total conductivity, by complex impedance, we can discriminate the contributions of grains and grain boundaries, as well as the interface between electrodes and electrolytes. The typical impedance spectra of an electrolyte material in the form of Nyquist plots (as the negative of an imaginary component of impedance (–Z_imag_) versus the real component of impedance (Z_real_)) consists of three successive circular semicircles in different frequency ranges. The number of semicircles that can be formed depends on the nature of the electrolyte, temperature, and the frequency range expansion. The semicircles at high and intermediate frequencies are described to bulk and grain boundary processes, respectively, while the semicircle at low frequency represents the electrode process contribution [26]. All these processes could be described using equivalent electrical circuits [26,27]. The high-frequency semicircle is usually attributed to the parallel connection of the bulk resistance of crystallite grains Rb and the geometric capacitance Cg. In this case, the high-frequency semicircle maximum (ω_max_,_b_) is calculated according to the equation ω_max,b_ = 1/R_b_•C_g_. On the other hand, the low-frequency semicircle is usually attributed to the grain boundary resistance R_gb_ in parallel connection with the intergranular capacitance C_gb_, and frequency was calculated by using equation ω_max,ig_ = 1/R_ig_•C_ig_.

**Figure 11:**
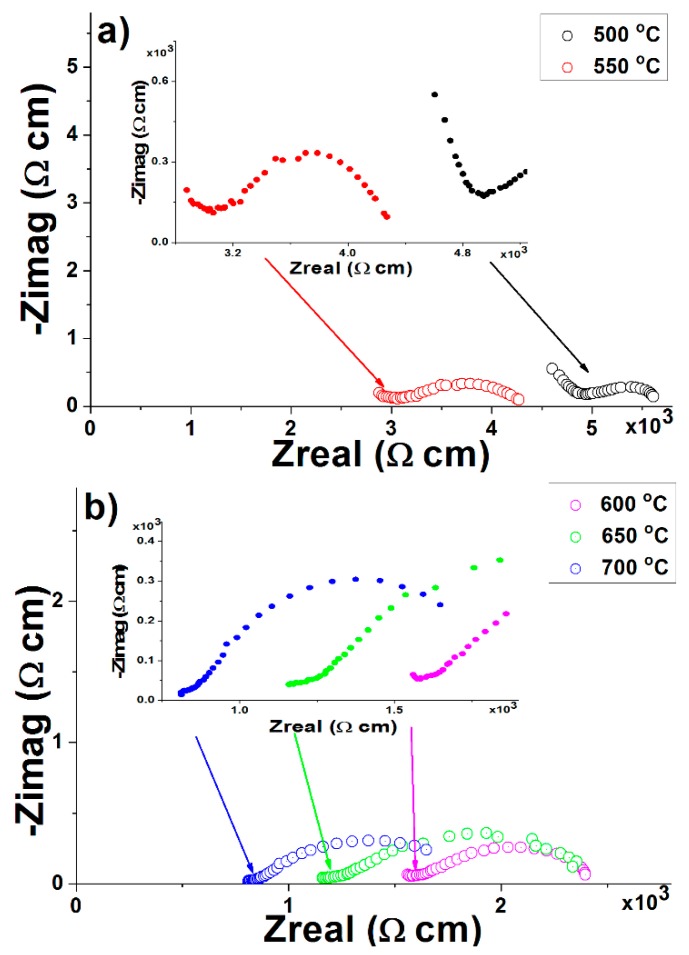
Complex impedance plots of GP_CeO_2__MGNP sample measured in different temperature ranges: (**a**) from 500 to 550 °C; (**b**) from 600 to 700 °C, in an air atmosphere. The arrows indicate the points on the real axis corresponding to the readings R_b_ + R_gb_.

In this study, the electrical conductivity of all the prepared GP_CeO_2_ samples have been measured as solid electrolytes in the temperature range of 500–700 °C with the increments of 50 °C, in order to determine their potential application in IT-SOFCs. All the results of electrical conductivity were determined from the original Nyquist plots recorded in the available frequency range of 1–100 kHz, and the results are presented in Table 1. As an example, which illustrated impedance behavior, the Nyquist plots of the sample GP_CeO_2__MGNP, which showed the highest value of total electrical conductivity (Table 1), is presented in Figure 11. Usually in the high-impedance semicircles, which were clearly separated (R_b_C_g_ << R_gb_C_gb_), the values of R_b_ and R_gb_ may be determined separately as a low-frequency intercepts of the semicircles with the real axis. As it is shown from the Figure 11, both resistance elements (R_b_ and R_gb_) decrease with the increase in temperature, which caused an increase in ω_max_. Consequently, the whole region of the impedance points shifts toward the low-frequency semicircle. Thus, at higher temperatures, the time constants associated with the bulk and grain boundary impedances are much lower than those associated with the electrode interface. In this case, semicircles due to bulk and grain boundary disappear, and only a single semicircle due to electrode interfacial processes can be observed [26,27]. Therefore, only the whole sum R_b_ + R_ig_ became readable, and the values of total resistance were estimated from the experimental cross-section of obtained semicircles with the real component of impedance (Z_real_). This intercept is marked by the arrows in Figure 11 (inset). Namely, the molecular structure of geopolymers is described as a matrix of alumina and silica tetrahedra joined at the corners with oxygen [28], where molecules of the water and hydroxyl group present the most important factors that influence electrical conductivity at room temperature. However, this effect can be eliminated as a common characteristic of thermal behavior of all geopolymers that undergo dehydratation and dehydroxylation reactions. The mentioned reactions of dehydratation and dehydroxylation lead to bond tetrahedral and octahedral by mutual oxygen atom (confirmed by FTIR analysis). By thermal treatment at 750 °C, the structural transformation of geopolymers occurs, and it transforms into a high-temperature mixed ceramics nNa_2_O·Al_2_O_3_·xSiO_2_·yH_2_O with CeO_2_ in the structure. In this case, ion conductivity in these materials occurs mainly via O interstitials. In addition, the incorporation of CeO_2_ in micro and mesopores obtained conductive pathways for O^2−^ ions from CeO_2_ as a new solid phase in the geopolymer structure. The better packing in micropores and mesopores of GP and lower inner activity of uniform particles CeO_2_ obtained by MGNP and SPRT enabled forming a dense conductive geopolymer/CeO_2_ structure. That obtained dense geopolymer stabile structures (according to DTA/TGA and SEM methods), which have an essential role in the formation of the microgalvanic couples necessary for the transport of ions and electrons in ion-electron processes. Electrons and ions exchange, and current flow through discrete energy bands, occur in the form of a conductive pathway between CeO_2_ as active centers. Therefore, conduction in such active geopolymers occurs through formed solid phases by localization in form micromorphology secondary changes from CeO_2_. This fact is exactly the reason for the significant increase of the conductivity of a geopolymer doped by CeO_2__MGNP. The electrode/electrolyte contribution to the overall electrolyte resistance has not been considered in this work, because, as noted above, the total resistance of electrolyte is given by the sum of grains (R_g_) and grain boundaries resistance (R_gb_). A new semicircle is highly visible in a low-frequency region in the temperature range of 600–700 °C (Figure 11b); this almost doubtless originates from the oxygen electrode reactions, O_2_/O^2−^ [27], which is not within the scope of this study.

The total electrical conductivity of the GP_CeO_2_ samples is shown in Table 1. The highest conductivity was determined for the sample GP_CeO_2__MGNP, amounting at 700 °C to 1.86 × 10^−2^ Ω^−1^ cm^−1^. Comparing the results obtained for electrical conductivity with the literature data [6,14,25,26,27], it can be noted that the values obtained in this study are higher. More specifically, the values of conductivity observed at 700 °C in this study are similar with literature values for 800 and 900 °C [6,14,25,26,27]. The same case is seen when comparing obtained results at low temperatures with literature data [6,14,25,26,27]. However, it is interesting to note that here-applied synthesis procedure offers benefits regarding better control of the chemical reaction and the precise stoichiometry of the final product. In addition, these synthesis methods are very reliable, while the equipment needed is extremely simple and inexpensive. In order to identify strategies for the optimization of the conductivities, the suggestion is to use a wide range of doping geopolymer systems by La or Gd [25,26,27], due to the higher conductivities of this system in comparison with samples containing smaller rare earths elements. It should be especially emphasized that the significant improvements in conductivity are possible, which would lead to the potential application of geopolymer-type electrolytes in fuel cells.

**Figure 12:**
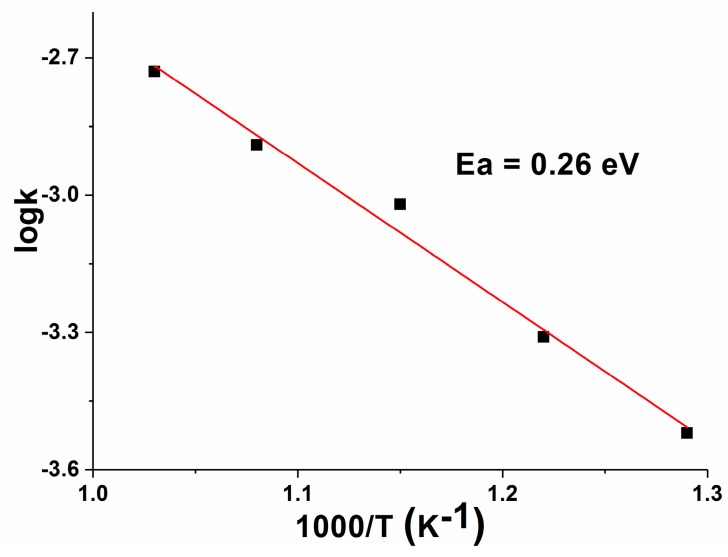
The dependence log κ = f(1/T) of the GP_CeO_2__MGNP sample.

According to the results listed in Table 1, the dependence log κ = f(1/T) of the sample GP_CeO_2__MGNP is presented in Figure 12. The activation energy of conductivity (E_a_) was calculated from the Arrhenius plot according to the equation ln(σ•T) = lnA-E_a_/k•1/T, where σ is the conductivity, T is the absolute temperature, A is the pre-exponential factor, and k is the Boltzmann constant. The activation energy of total conductivity for the GP_CeO_2__MGNP sample was 0.26 eV. Based on the data found in the literature [6,14,25,26,27], it can be concluded that the value of E_a_ presented in this study is much lower. In addition, comparing the obtained values of E_a_ with the activation energy of similar oxygen ion conductors containing dopants [25,26,27], the E_a_ values observed in this study are significantly lower. This is a consequence of the well-ordered structure and better processability of geopolymer–CeO_2_ materials.

The presented electrical characteristics of the GP_CeO_2__MGNP materials indicate the superiority of solid solution for the application in IT-SOFCs. Bearing in mind that a low temperature necessary for obtaining samples, it can be concluded that the obtained material is characterized by high stability, which is important for its potential use as a solid electrolyte. However, it is important to note that the future research should encompass further work on routes molecular dynamics (MD) simulations of conduction mechanisms and the general search for improved conductors with increases in the stability system IT-SOFCs electrolyte/electrode. Additionally, the breadth of possible doping regimes in this material provides new opportunities to design and optimize their transport parameters for potential applications in IT-SOFC as clean energy.

## 4. Conclusions

For the first time, new composite materials were successfully obtained from alkali-activated metakaolin, in the presence of 10% differently synthesized CeO_2_ powders (GP_CeO_2__com, GP_CeO_2__MGNP, and GP_CeO_2__SPRT), as solid electrolytes for application in IT-SOFC. The detailed structural XRPD and MALDI-TOF analysis confirmed the formation of a solid CeO_2_ phase, whose particles allow electrochemical activation of the obtained samples. The DTA-TGA analyses confirmed the thermal stability of all the GP_CeO_2_ samples. The BET, FE-SEM, and EDS results indicated that particles of CeO_2_ were stabile interconnected and form a continual conductive path, which was confirmed by the complex impedance method (EIS) method. The highest conductivity was determined for the sample GP_CeO_2__MGNP (1.86 × 10^−2^ Ω^−1^ cm^−1^ at 700 °C) with an activation energy of 0.26 eV in the temperature range of 500–700 °C. The achieved value of electrical conductivity of the GP_CeO_2__MGNP indicates the advantage of this material as a solid electrolyte and its opportunity to be designed and optimized for the application in IT-SOFCs.

## Figures and Tables

**Table 1:** The temperature dependence of total electrical conductivity (κ) of the pure GP, GP_CeO_2__com, GP_CeO_2__MGNP, and GP_CeO_2__SPRT.

COMPOSITION	κ (Ω^−1^ cm^−1^) 500 °C	κ (Ω^−1^ cm^−1^) 550 °C	κ (Ω^−1^ cm^−1^) 600 °C	κ (Ω^−1^ cm^−1^) 650 °C	κ (Ω^−1^ cm^−1^) 700 °C
GP	2.34 × 10^−3^	3.33 × 10^−3^	5.35 × 10^−3^	9.32 × 10^−3^	1.04 × 10^−2^
GP_CeO2_com	2.31 × 10^−3^	3.02 × 10^−3^	2.34 × 10^−3^	5.29 × 10^−3^	9.89 × 10^−3^
GP_CeO2_MGNP	2.39 × 10^−3^	3.52 × 10^−3^	5.48 × 10^−3^	9.53 × 10^−3^	1.86 × 10^−2^
GP_CeO2_SPRT	3.89 × 10^−3^	6.09 × 10^−3^	1.03 × 10^−2^	2.84 × 10^−2^	5.52 × 10^−3^
